# Beyond D’Amico risk classes for predicting recurrence after external beam radiotherapy for prostate cancer: the Candiolo classifier

**DOI:** 10.1186/s13014-016-0599-5

**Published:** 2016-02-24

**Authors:** Domenico Gabriele, Barbara A Jereczek-Fossa, Marco Krengli, Elisabetta Garibaldi, Maria Tessa, Gregorio Moro, Giuseppe Girelli, Pietro Gabriele, the EUREKA-2 consortium

**Affiliations:** Neuroscience Department, Human Physiology Section, University of Torino, corso Raffaello 30, 10125 Torino, Italy; Division of Radiation Oncology, European Institute of Oncology, and University of Milan, Milan, Italy; Division of Radiation Oncology, Maggiore University Hospital of Novara, Novara, Italy; Division of Radiation Oncology, FPO-IRCCS Cancer Center of Candiolo (Torino), Candiolo, Italy; Division of Radiation Oncology, Cardinal Massaia Hospital, Asti, Italy; Division of Radiation Oncology, degli infermi Hospital, Biella, Italy; Division of Radiation Oncology, Civile Hospital, Ivrea, Italy

**Keywords:** Prostate cancer, Radiation therapy, Predictive modelling, Risk classification, Outcome

## Abstract

**Background:**

The aim of this work is to develop an algorithm to predict recurrence in prostate cancer patients treated with radical radiotherapy, getting up to a prognostic power higher than traditional D’Amico risk classification.

**Methods:**

Two thousand four hundred ninety-three men belonging to the EUREKA-2 retrospective multi-centric database on prostate cancer and treated with external-beam radiotherapy as primary treatment comprised the study population. A Cox regression time to PSA failure analysis was performed in univariate and multivariate settings, evaluating the predictive ability of age, pre-treatment PSA, clinical-radiological staging, Gleason score and percentage of positive cores at biopsy (%PC). The accuracy of this model was checked with bootstrapping statistics. Subgroups for all the variables’ combinations were combined to classify patients into five different “Candiolo” risk-classes for biochemical Progression Free Survival (bPFS); thereafter, they were also applied to clinical PFS (cPFS), systemic PFS (sPFS) and Prostate Cancer Specific Survival (PCSS), and compared to D’Amico risk grouping performances.

**Results:**

The Candiolo classifier splits patients in 5 risk-groups with the following 10-years bPFS, cPFS, sPFS and PCSS: for very-low-risk 90 %, 94 %, 100 % and 100 %; for low-risk 74 %, 88 %, 94 % and 98 %; for intermediate-risk 60 %, 82 %, 91 % and 92 %; for high-risk 43 %, 55 %, 80 % and 89 % and for very-high-risk 14 %, 38 %, 56 % and 70 %. Our classifier outperforms D’Amico risk classes for all the end-points evaluated, with concordance indexes of 71.5 %, 75.5 %, 80 % and 80.5 % versus 63 %, 65.5 %, 69.5 % and 69 %, respectively.

**Conclusions:**

Our classification tool, combining five clinical and easily available parameters, seems to better stratify patients in predicting prostate cancer recurrence after radiotherapy compared to the traditional D’Amico risk classes.

**Electronic supplementary material:**

The online version of this article (doi:10.1186/s13014-016-0599-5) contains supplementary material, which is available to authorized users.

## Background

Prostate cancer (PCa) is the most common cancer in men and the second most common cause of death from tumors in males [1]. Several nomograms have been developed to guide therapy and predict outcome following radiotherapy: one of the most popular classifications is the D’Amico risk classifier, dividing patients according to pre-treatment PSA, clinical stage and biopsy-based Gleason score (bGS) into three categories: low-risk (PSA <10 ng/ml and cT1-cT2a and bGS ≤6) , intermediate-risk (PSA 10–20 ng/ml or cT2b or bGS 7) and high-risk (PSA >20 ng/ml or clinical stage ≥ cT2c or bGS ≥8) [2].

Several additional pathological, clinical-radiological and molecular markers have been studied in the past 15 years to implement D’Amico classification [3]: of these, the simplest and more readily available information are age at treatment and the percentage of positive biopsy cores (%PC). In 2000–2001, D’Amico reported the independent prognostic ability of %PC to foresee biochemical recurrence beyond traditional risk factors in a radical prostatectomy cohort of 823 men [4] and in 473 men treated by external-beam radiotherapy (EBRT) [5]. Regarding age, Siddiqui [6] reported in a radical prostatectomy cohort of 5509 men a higher risk of systemic progression and death from prostate cancer in patients younger than 55 compared to patients older than 70. In addition, Ribeiro [7] confirmed that age lower than 60 predicts for a poor outcome (time to progression and overall survival) in metastatic prostate cancer receiving androgen deprivation therapy (ADT).

In our study, based on a large database of thousands of patients affected by prostate cancer and treated with EBRT, we developed a 5-classes nomogram for predicting biochemical Progression Free Survival (bPFS) including not only pre-treatment PSA, stage and bGS, but also age and %PC. Thereafter, we applied it to predict clinical PFS (cPFS), systemic PFS (sPFS) and Prostate Cancer Specific Survival (PCSS) too, and compared the performance of our nomogram with the D’Amico risk classification.

## Methods

### Patient population

Two thousand four hundred ninety three men affected by prostate cancer and treated with EBRT as primary treatment comprised the study population.

This cohort belongs to the EUREKA-2 retrospective multi-centric database, including 3776 cases of radio-treated prostate cancer cases in North-West Italy between 1997 and 2012, approved by FPO-IRCCS Cancer Center of Candiolo Ethical Committee in July 2013 and amended in November 2014. Ten Radiotherapy Divisions participated to EUREKA-2 study, seven of which in Piedmont Italian region, two in Lombardy and one in Tuscany (full information are provided in Additional file 1: Table S1). Inclusion criteria for EUREKA-2 study were: histologic diagnosis of adenocarcinoma of the prostate; Radical Radiotherapy as first-line treatment, performed with conformational technique within the period January 1^st^, 1997 to December 31^st^, 2012; at least two of the following three pre-treatment parameters: PSA, staging, Gleason Score; case histories, clinical and serological follow-ups available for clinical data collection. Concerning privacy procedures, data were pseudo-anonymized by local researchers within the participating Hospitals. The two lists with patients’ personal data and clinical data were kept separated and only clinical data, and not personal data, were sent to EUREKA-2 common database. Consequently, outside the hospitals and in the common EUREKA-2 database data are anonymous and by no way amenable to the single patient identity. The files are stored with Microsoft Excel and Microsoft Access data management software (®MicroSoft Corporation, Redmond, Washington, USA) according to different domains: socio-demographic group of data, biopsy and staging, therapies, oncologic outcomes, serologic follow-up and collateral effects.

In particular, from the whole EUREKA-2 database were excluded 1283 patients without complete information regarding established pre-treatment factors (PSA, clinical-radiologic stage and bGS) and the number of total and positive biopsy cores.

### Preoperative staging and treatment

In all cases, staging evaluation included anamnesis, physical exam with Digital Rectal Examination (DRE), serum PSA and a trans-rectal ultrasound (TRUS) guided needle biopsy of the prostate with GS histologic grading. Radiological examinations (abdominal CT, endo-coil or pelvic MRI and bone scan) were performed according to the patient risk-class, to the physician’s opinion and to the available hospital facilities.

PSA was obtained before biopsy and radiologic studies. Primary, secondary and total GS were mainly attributed according to ISUP 2005 revised Gleason Score system [8]. The clinical-radiologic stage was obtained integrating the clinical AJCC 2010 staging system [9] with all the radiologic information available, while biopsy data were not taken into account. Age at treatment was calculated as the difference between the first day of radiotherapy and the date of birth, rounded to the closest integer number. The %PC was calculated multiplying 100 by the number of positive cores containing prostate cancer, of any length, and dividing by the total number of cores sampled.

All patients were treated with curative 3-Dimensional Conformal EBRT (3D-CRT) or Intensity Modulated Radiation Therapy (IMRT). The fractionation schedules to prostate-GTV (Gross Tumor Volume) varied between traditional fractionation of 1.8-2 Gy per fraction to moderate hypo-fractionation of 2.5-2.7 Gy per fraction; all doses were normalized to Equivalent Dose at 2 Gy per fraction (ED2Gy) using a mean α/β of 2.5 Gy for prostate cancer (according to literature α/β ratio for prostate cancer ranges between 1.5 and 5.7 Gy [10–12]). Treatment consisted of radiotherapy alone or radiotherapy combined with ADT in 38 % and 62 % of the cases, respectively.

### Follow-up

Median follow-up of the 2493 patients was 50 months. Standard follow-up included PSA and DRE every 3-months for 2 years, every 6-months until the fifth year and annually thereafter.

During the follow-up 453 patients (18 %) had a biochemical relapse, 249 (10 %) relapsed clinically, 138 (5.5 %) had distant metastases, and 233 (9 %) died, 72 of these (3 % of the total) because of prostate cancer.

Time 0 was defined as the last day of EBRT for all patients and PSA failure according to Phoenix consensus definition (i.e. a rise by 2 ng/mL or more above the nadir PSA [13]). Clinical relapse was defined as a recurrence in the prostate bed, regional lymph nodes or distant metastasis shown by radiologic examinations (bone scan, choline-PET-CT, MRI, CT, ultrasound) or by physical examination or by biopsy. Systemic relapse was defined as a distant metastasis, including bone or other visceral organs, shown by radiologic examinations or by physical examination. Prostate cancer specific mortality was defined as death because of prostate cancer, checked by a physician through patients’ case history reports, cancer regional registries and, if necessary, phone calls to the patient or to a close relative or General Practitioner of him (if the patient was dead).

### Statistical analyses

A Cox regression time to PSA failure analysis was performed in univariate and multivariate settings, evaluating the predictive ability of age, pre-treatment PSA, clinical-radiological stage, GS and %PC; besides, the regression algorithm was adjusted for RT dose (as a continuous variable) and combined therapy (RT + ADT or RT alone, as a dichotomous variable). The assumptions of the Cox model were tested and met.

All variables were evaluated as categorical variables: age ≥70 years or age <70 years; PSA <7 ng/ml, 7–15 ng/ml, or >15 ng/ml; clinical-radiologic stage cT1, cT2 or cT3-cT4; bGS ≤6, 3 + 4, 4 + 3, 8 or 9–10; %PC 1-20 %, 21-50 %, 51-80 % or 81-100 %.

Effects cell coding (i.e. 1 or 0 or −1 coding) was applied to the stratified variables, in order to calculate the relative Hazard Ratios (HRs) of the multivariate analysis compared to the outcome of the mean of the cohort. The accuracy of the model was checked with bootstrapping statistics (2493 patients, resampling with 1000 cases each replication for a total of 10,000 replications).

A 360-cells-table was built by multiplying the HRs of the subgroups for all the variables’ combinations; the following combined HRs were classified into 5 different risk classes for biochemical relapse: very-low-risk for HRs 0.17-0.30; low-risk for HRs 0.31-0.55; intermediate-risk for HRs 0.56-1.20; high-risk for HRs 1.21-2.40; and very-high-risk for HRs 2.41-6.60. In addition, an equivalent nomogram was built multiplying 100 by the beta coefficients associated to the HRs, normalizing each reference value of the variables to 0 points (i.e. age ≥70, PSA <7 ng/ml, stage cT1, bGS ≤6 and %PC 1-20 %), and summing up the values of the 5 prognostic factors into a scale ranging in between 0 and 363 total points, corresponding to the previously defined 5 risk classes.

Kaplan-Meier survival curves for bPFS, divided according to our 5-risk-classes and to D’Amico risk classes (for comparison), were graphed, overall and paired log-rank tests were performed and Concordance Indexes calculated. In addition, D’Amico risk classification and our 5-groups classification tool were further analyzed for cPFS, sPFS and PCSS and their prediction performances compared.

All statistical analyses were performed with Stata SE 13.1 Software (®StataCorp, Texas, USA).

## Results

The stage and treatment characteristics of the 2493 patients are listed in Table 1.

**Table 1:** Characteristics of our series of 2493 patients

Features	
Follow-up, months	
Mean (SD)	56 (36)
Median (Min-Max)	50 (4–159)
FU ≥ 2-yy, no (%)	2018 (81 %)
FU ≥ 5-yy, no (%)	982 (39 %)
FU ≥ 7-yy, no (%)	481 (19 %)
FU ≥ 10-yy, no (%)	179 (7 %)
Age, yr	
Mean (SD)	71.7 (5.9)
Median (Min-Max)	73 (43–86)
PSA, ng/ml	
Mean (SD)	15.0 (26.0)
Median (Min-Max)	8.6 (0.39-749)
Tumor Stage, %	
cT1	30.5 %
cT2	57.5 %
cT3-4	12 %
Bone Scan staging	
Performed	67 %
Not performed	33 %
Abdominal CT staging	
Performed	59 %
Not performed	41 %
Endo-coil or pelvic MRI staging	
Performed	15 %
Not performed	85 %
TRUS	
Performed	49 %
Not performed	51 %
Biopsic Gleason Score, %	
≤6	48 %
3 + 4	22 %
4 + 3	11.5 %
8	12 %
9-10	6.5 %
D’Amico Risk Classification, %	
Low	21.5 %
Intermediate	32 %
High	46.5 %
Biopsy cores sampled, no	
Mean (SD)	10.3 (4.2)
Median (Min-Max)	10 (2–42)
% Positive Cores, %	
Mean (SD)	44.3 % (28.0 %)
Median (Min-Max)	40 % (3-100 %)
RT Dose, ED2Gy	
Mean (SD)	75.5 (3.0)
Median (Min-Max)	76.0 (67.1-81.1)
RT alone, %	38 %
RT plus ADT, %	62 %

*SD* Standard deviation, *TRUS* Trans rectal ultra-sound, *ED2Gy* Equivalent dose at standard 2 Gy dose per fraction, *RT* Radiation therapy, *ADT* Androgen deprivation therapy

All variables evaluated, i.e. age, pre-treatment PSA, clinical-radiologic stage, bGS and %PC are highly significant predictors of biochemical relapse in both univariate and multivariate Cox models: all Ps are lower than 0.001 except for age, whose values are *P* = 0.001 and *P* = 0.019 in univariate and multivariate analyses, respectively (Table 2). In particular, the risk of recurrence rises in younger patients and increases gradually with higher PSA, wider clinical-radiologic extension in/out prostate, higher bGS and a higher percentage of biopsy cores affected by cancer. Internal validation performed with bootstrapping shows a good reliability of the model as a whole: PSA and bGS remain highly significant (*P* < 0.001 and *P* = 0.012, respectively), %PC and clinical-radiologic stage are significant (*P* = 0.008 and *P* = 0.031), while age shows a trend but loses its statistical significance (*P* = 0.16; see Table 2 last column).

**Table 2:** Univariate and multivariate cox regression (time to PSA failure) and bootstrapping analysis

Variables	Sub-groups	Univariate analysis		Multivariate analysis		Bootstrapping
		HR	*P* value	HR	*P* value	*P* value
Age	≥70 yy	0.85	0.001	0.89	0.019	0.16
	<70 yy	1.18		1.12		
PSA	<7	0.52	<0.001	0.63	<0.001	<0.001
	7-15	0.88		0.96		
	>15	2.17		1.65		
Staging	cT1	0.49	<0.001	0.77	<0.001	0.031
	cT2	0.95		0.93		
	cT3-4	2.17		1.40		
bGS	≤6	0.47	<0.001	0.59	<0.001	0.012
	3 + 4	0.77		0.83		
	4 + 3	0.99		0.95		
	8	1.31		1.26		
	9-10	2.13		1.70		
% Positive Cores	1-20 %	0.48	<0.001	0.67	<0.001	0.008
	21-50 %	0.76		0.89		
	51-80 %	1.22		1.11		
	81-100 %	2.26		1.50		
RT Dose	Continuous	0.88 / Gy	<0.001	0.89 / Gy	<0.001	0.001
Therapy schedule	RT alone	0.81	<0.001	1.25	<0.001	0.028
	RT & ADT	1.24		0.80		

**Fig. 1:**
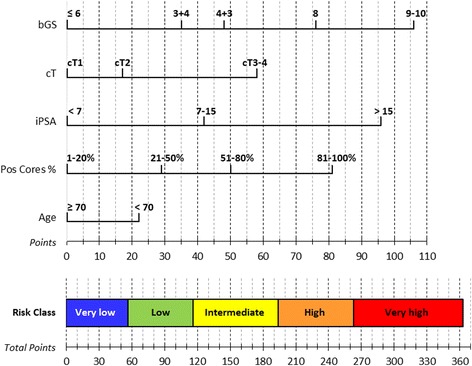
Candiolo nomogram. Points: bGS ≤ 6 0 pt, bGS = 3 + 4 35 pt, bGS = 4 + 3 48 pt, bGS = 8 76 pt, bGS = 9-10 106 pt; cT1 0 pt, cT2 17 pt, cT3-4 58 pt; PSA < 7 0 pt, PSA7-15 42 pt, PSA > 15 96 pt; %PC 1-20 % 0 pt, 21-50 % 29 pt, 51-80 % 50 pt, 81-100 % 81 pt; age ≥ 70 0 pt, age < 70 22 pt; very-low risk 0–56 pt, low risk 57–116 pt, intermediate risk 117–193 pt, high risk 194–262 pt, very-high risk 263–363 points

The 360-cells-table combining all the possible combinations of the stratified parameters clearly shows a strong trend, going from very-low risk (in blue) on the upper-left corner to very-high-risk (in red) in the lower-right corner; in between can be noticed low-risk (in green), intermediate-risk (in yellow) and high-risk (in orange, see Table 3 and Additional file 1: Table S2). Very-low-risk group includes 529 patients (21 %), low-risk 770 (31 %), intermediate risk 696 (28 %), high-risk 329 (13 %) and very-high risk 169 (7 %); full data on patients’ distribution according to model parameters are illustrated in (Additional file 1: Table S3). Besides, the related Candiolo nomogram is displayed in Fig. 1.

**Table 3:**
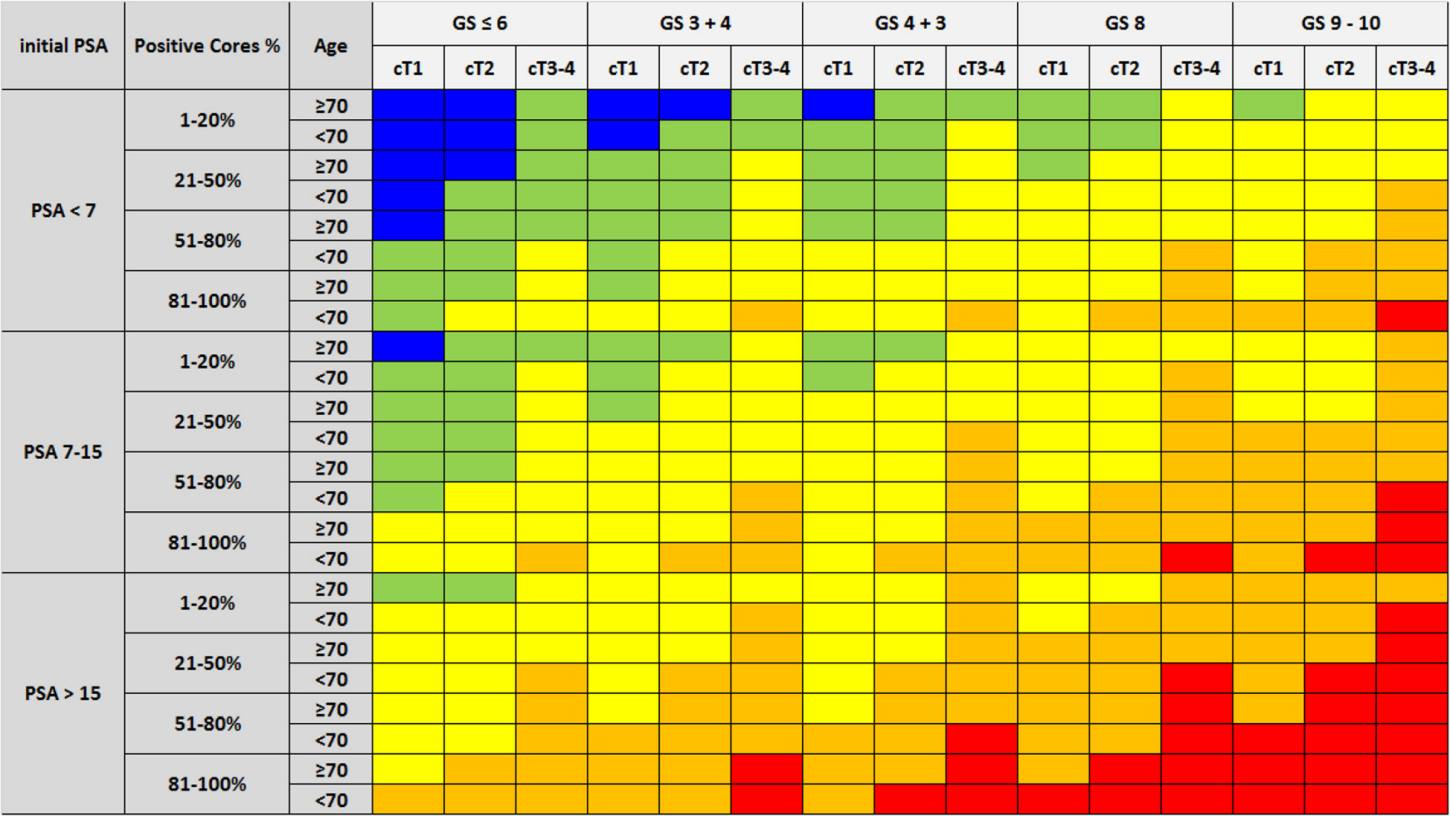
Candiolo classifier table: very-low-risk blue, low-risk green, intermediate-risk yellow, high-risk orange, very-high-risk red

**Fig. 2:**
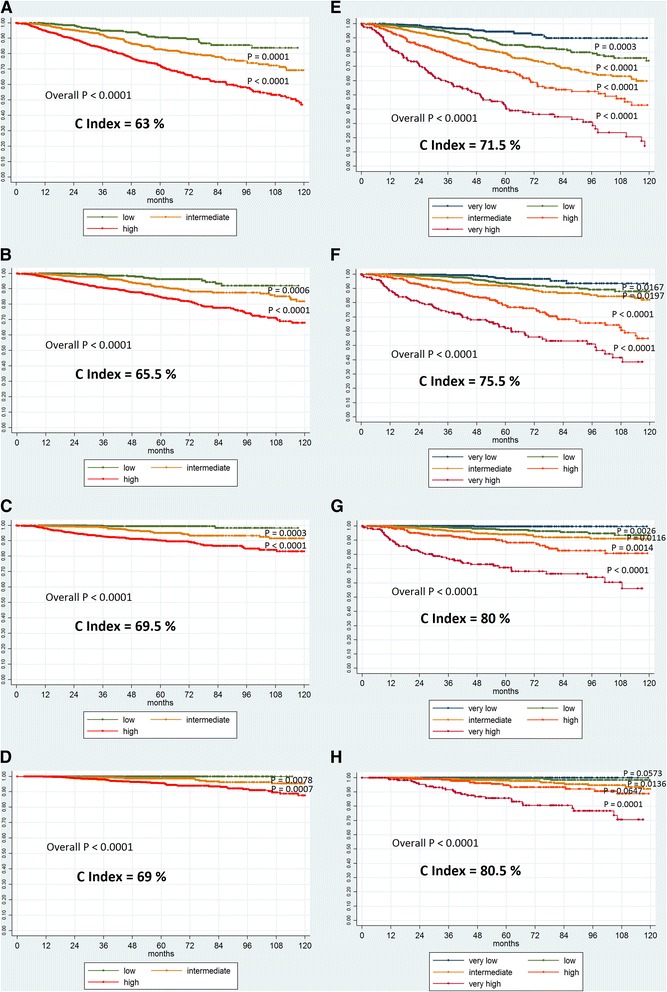
bPFS (a-e), cPFS (b-f), sPFS (c-g) and PCSS (d-h) according to D’Amico classification (a-b-c-d) or to Candiolo classifier (e-f-g-h). Kaplan-Meier curves with overall and paired log-rank-test results, and Concordance Indexes in bold

In Fig. 2 are shown the Kaplan-Meier curves for bPFS (a-e), cPFS (b-f), sPFS (c-g) and PCSS (d-h) according to D’Amico risk classification (a-b-c-d) or to Candiolo classifier (e-f-g-h) with overall and paired log-rank-test results. The Concordance Indexes for Candiolo nomogram are 71.5 %, 75.5 %, 80 % and 80.5 % for bPFS, cPFS, sPFS and PCSS, respectively, consistently higher than D’Amico ones (63 %, 65.5 %, 69.5 % and 69 %, respectively).

In addition, Table 4 resumes yearly (until 10 years of follow-up) bPFS, cPFS, sPFS and PCSS for the five-classes of the Candiolo classifier. In particular, bPFS ranges at 5-yy between 94 % (very-low-risk) and 43 % (very-high-risk) and at 10-yy between 90 % and 14 %; cPFS varies at 5-yy between 97 % and 62 % and at 10-yy between 94 % and 38 %; sPFS ranges at 5-yy between 100 % and 71 % and at 10-yy between 100 % and 56 %; PCSS ranges at 5-yy between 100 % and 86 % and at 10-yy between 100 % and 70 %.

**Table 4:** Life tables: bPFS, cPFS, sPFS and PCSS for the five-classes of the Candiolo classifier

Candiolo risk class	Biochemical PFS – follow-up (years)									
	1	2	3	4	5 yy	6	7	8	9	10 yy
Very-Low	99 %	99 %	96 %	95 %	94 %	92 %	90 %	90 %	90 %	90 %
Low	99 %	97 %	94 %	90 %	85 %	84 %	82 %	80 %	76 %	74 %
Intermediate	98 %	94 %	90 %	83 %	80 %	74 %	69 %	65 %	63 %	60 %
High	95 %	85 %	78 %	71 %	67 %	60 %	54 %	52 %	47 %	43 %
Very-High	83 %	72 %	60 %	51 %	43 %	36 %	35 %	31 %	23 %	14 %
Candiolo Risk Class	clinical PFS – Follow-up (years)									
	1	2	3	4	5 yy	6	7	8	9	10 yy
Very-Low	100 %	100 %	99 %	99 %	97 %	97 %	96 %	94 %	94 %	94 %
Low	99 %	99 %	98 %	96 %	94 %	92 %	91 %	89 %	88 %	88 %
Intermediate	99 %	97 %	95 %	93 %	92 %	89 %	87 %	85 %	85 %	82 %
High	98 %	93 %	89 %	84 %	79 %	76 %	68 %	66 %	63 %	55 %
Very-High	89 %	81 %	74 %	68 %	62 %	56 %	53 %	51 %	42 %	38 %
Candiolo Risk Class	systemic PFS – Follow-up (years)									
	1	2	3	4	5 yy	6	7	8	9	10 yy
Very-Low	100 %	100 %	100 %	100 %	100 %	100 %	100 %	100 %	100 %	100 %
Low	100 %	100 %	99 %	98 %	97 %	97 %	96 %	95 %	94 %	94 %
Intermediate	100 %	98 %	97 %	95 %	94 %	93 %	92 %	91 %	91 %	91 %
High	99 %	95 %	93 %	90 %	89 %	88 %	82 %	82 %	80 %	80 %
Very-High	90 %	82 %	77 %	73 %	71 %	68 %	66 %	64 %	60 %	56 %
Candiolo Risk Class	PCSS – Follow-up (years)									
	1	2	3	4	5 yy	6	7	8	9	10 yy
Very-Low	100 %	100 %	100 %	100 %	100 %	100 %	100 %	100 %	100 %	100 %
Low	100 %	100 %	99 %	99 %	99 %	99 %	98 %	98 %	98 %	98 %
Intermediate	100 %	100 %	99 %	98 %	98 %	98 %	96 %	95 %	95 %	92 %
High	100 %	99 %	99 %	96 %	95 %	93 %	93 %	92 %	89 %	89 %
Very-High	99 %	95 %	92 %	87 %	86 %	80 %	80 %	76 %	70 %	70 %

## Discussion

The Candiolo classifier differs significantly from the D’Amico risk classification. As far as pre-treatment PSA is concerned, D’Amico advises the cut-offs of 10 and 20 ng/ml, while our optimal boundaries are slightly lower: 7 and 15 ng/ml. Probably the difference relies in the cohort, our whole patients pertaining to post-PSA screening era; but also in the introduction of percentage of positive cores as a covariate, carrying a second information about cancer extension and therefore able to shift the best stratification cut-offs.

Concerning bGS, we definitely refined the traditional coarse classification in 3-classes (≤6, 7 or ≥8) dividing patients according to a more precise 5-classes layering, in particular discerning 3 + 4 versus 4 + 3 patterns and 8 versus 9–10 ones. Taking ISUP 2005 modified Gleason Score as a time cut-off [8], i.e. comparing patients treated up to 2004 (569 cases) versus 2005 onwards (1924 patients), there is an almost significant time trend concerning total biopsic Gleason Score with bGS means increasing from 6.5 to 6.7, respectively (both medians are equal to 7, *P* = 0.06 at Wilcoxon-Mann–Whitney test). However, we think this trend does not affect substantially our nomogram because of three main reasons: the difference in absolute terms is quite small, the number of patients treated prior to ISUP 2005 Gleason Score revision is only 23 % of the total, and finally we reduced the under-grading influence in older cases (in which total bGS of 2-3-4 were much more frequent) by combining together all bGS ≤ 6 into a single risk category.

Considering staging, the D’amico clinical staging, splitting patients according to their cT2 stage (≤cT2a, cT2b or ≥ cT2c) was replaced by a clinical and radiological staging taking advantage of radiologic information and dividing patients according to more objective criteria: cT1 microscopic cancer, cT2 macroscopic but intra-prostatic cancer, cT3-4 macroscopic and extra-prostatic adenocarcinoma. Actually, Epstein [14] found, even if in a radical prostatectomy cohort, that pT2b staging almost doesn’t exist and that data are conflicting about the power of pT2 disease sub-staging to further stratify patients. According to Algarra [15] MRI data optimize D’Amico risk groups in predicting bPFS after radical prostatectomy. Unfortunately, only 15 % of our patients were locally staged through MRI (we calculated with logistic regression a strong association between MRI staging and T3 with an OR = 6.98 and a *P* < 0.001). However, this relationship may be over-estimated because of a selection bias (MRI is performed usually in suspect of extra-prostatic extension) and it may be partially compensated by the widespread execution of CT and ultra-sound in 59 % and 49 % of our patients, respectively; precisely, the percentage of patients having neither CT nor MRI for loco-regional staging decreases in our cohort to 35 %.

In addition, we added %PC and age in the prognostic algorithm. The prognostic significance of tumor volume has been tested both in radical prostatectomy and needle biopsy specimens. Therefore, it is recommended to record at least the number of positive and total cores along with one other more detailed measurement such as the percent of the core involvement or length of cancer [14]. Besides, in 2007 Briganti [16] showed that %PC can improve the ability to predict lymph nodes invasion in patients undergoing radical prostatectomy and extended pelvic lymph node dissection: %PC and bGS were the most informative predictors of Lymph Node Involvement, overcoming PSA and stage. The addition of %PC to a nomogram including traditional risk factors was able to improve overall accuracy from 79.7 % to 83 % (*P* < 0.001). In the early 2000s, D’Amico clearly proved the independent value of positive prostate biopsies in predicting biochemical outcome after radical prostatectomy [4] or after EBRT [5]. The information about tumor extension at biopsy was clinically significant to better classify intermediate-risk patients; in both studies patients were stratified in three classes: %PC < 34 %, 34-50 %, %PC > 50 %. In 2004 D’Amico [17] further showed that %PC was superior to traditional risk factors in predicting prostate cancer specific mortality (PCSM) in patients with low or favorable intermediate-risk; the relative risk of PCSM for patients with %PC ≥ 50 % as compared with <50 % was 10.4. However, Kupka [18] reported in a cohort of 249 men treated with RP that even prostate cancer with single positive core is associated with considerable rates of over-grading for the pathological GS, pT2c-pT3 and positive surgical margin. Therefore, %PC should not be used alone to predict patients’ prognosis after radical therapy, and treatment plan has always to take into account the traditional prognostic factors, too. Therefore, several reports and our analysis strongly support the statistical significance of %PC as an additional prognostic factor of prostate cancer recurrence.

Literature is more scarce concerning age, showing for older patients a lower rate of distant metastases or prostate cancer deaths, but not an advantage in biochemical relapse [6, 7]. The finding is confirmed by our data, where the statistical significance of the variable age was the lowest between the selected prognostic factors of biochemical recurrence and failed the internal validation with bootstrapping. However, its relevance may be much more powerful for late end-points like sPFS and PCSS, and so this could be one of the determinant factors of the higher concordance indexes for these end-points by the Candiolo classifier (around 80 %). In fact, two main reasons can justify the worse prognosis of younger patients: intrinsic biologic differences of prostate cancer and variations in patient’s hormonal status at different ages, and a greater likelihood of experiencing progression or death since they are less likely to die of competing comorbidity conditions. We chose the 70 years cut-off according to a previous paper of our research group at FPO-IRCCS Cancer Center of Candiolo, published by Maggio et al. in 2012 [19]. It showed that patients older than 70 years have better outcomes compared with younger ones, and precisely that, at 90 months of follow-up, Overall Survival and Disease-Free Survival (biochemical and clinical) were 10 % and 16 % higher in older patients, respectively; besides, there was no significant difference in the distribution of pre-RT risk-classes between age groups. Moreover, the 70 years cut-off is clinically useful because next to the median and mean ages of our cohort, that are 73 and 71.7 years, respectively.

Our five classes are statistically different at paired comparisons for bPFS, cPFS, sPFS, while for PCSS three main groups may be identified: very-low and low-risk together (summing up to 52 % of the overall cohort) with almost no dead at 10 years, intermediate and high-risk with a specific mortality at 10-yy around 10 % and very-high-risk alone with a 10-yy mortality of 30 %.

The mean prostate cancer specific survival in our cohort at 5 and 10 years are 97.5 % and 92.5 %, respectively. These high values may be justified by the intense therapy, performed both as a first-line treatment (62 % of the cases where treated with the combination RT + ADT) and/or as a salvage or palliative therapy after biochemical and/or clinical recurrence (ADT alone 82 %, chemotherapy 3 %, local therapies i.e. surgery, RT, HIFU and cryotherapy 6 %, ADT plus local therapy 3 %, none 6 %).

The main strengths of our study are: the large numerosity of our sample (2493 patients), widely distributed according to risk factors combinations (e.g. PSA from <1 to >100 ng/ml), and with complete staging and treatment information; the integration of five significant prognostic factors according to all their possible combinations; the five-risk classification instead of three-risk-classes with wider prognostic differences; the Proportional Hazard assumption of the Cox model fulfilled (see in the Additional file 1: Figure S1); the internal validation with bootstrapping performed; the nomogram, developed with bPFS as setting end-point, was successfully applied to other three following outcomes, i.e. cPFS, sPFS and PCSS.

On the other hand, our study has several limitations, mainly related to its retrospective and multi-centric nature, and to the absence of an external validation. In particular, the biopsy schemes adopted by each center were heterogeneous and there wasn’t any central pathologic review (even if by a decade Piedmont Oncology Web fosters pathology quality assessment on prostate cancer through guidelines and periodic meetings [20, 21]). Furthermore, radiologic staging protocols changed in time with a low total number of MRI, as well as the RT prescription doses, fractionation schedules and the use of combined therapy with ADT; and median follow-up of 50 months is still short for a “slow killer” like prostate cancer. Besides, the model applied was a simple linear regression with no interaction factors considered: it allows the modelling to be simple and robust, but does not take into consideration more complex modelling. In addition, an external validation is needed as soon as possible.

## Conclusions

In conclusion, our study develops a new risk classification tool, that we called the “Candiolo classifier”, in a wide cohort of radio-treated patients at higher risk of relapse (46.5 % of our cases are in D’Amico high-risk group versus 21 % of D’Amico early 2000s studies [4, 5]) treated with higher doses of RT (median dose of 76 Gy versus 70.2 Gy of the D’Amico studies) and treated with RT + ADT in 62 % of cases. Besides, we subdivided our patients into five different classes instead of three, with Concordance Indexes around 10 % higher in foreseeing bPFS, cPFS, sPFS and PCSS.

We would like to evidence some points about our 5-classes distribution. First, even rare 3 + 4 or 4 + 3 cancers with all other positive factors may be included in very-low-risk group. Second, a quite high number of patients (21 %) can be classified in very-low risk class with 100 % sPFS and PCSS at 10 years, and can be of consequence selected for watchful waiting approach too. Third, only a restricted number of patients (7 % of the total) belong to very-high-risk class, with 30 % of prostate cancer specific deaths at 10 years.

We further recommend the development of a nomogram integrating in this subset of patients also therapeutic information (RT dose and ADT) and the enhancement of a tailored follow-up schedule according to the risk of recurrence.

## Additional file

Additional file 1:
**Table S1.** EUREKA-2 study, Radiotherapy participating centers. **Table S2.** Candiolo classifier table with Hazard Ratio combinations. **Table S3.** Candiolo classifier table with complete patients’ distribution. **Figure S1.** Graphical assessment of the Proportional Hazard assumption of the Cox model (observed data compared to predicted ones). (PDF 381 kb)

## Abbreviations

%PC: Percentage of positive cores at biopsy
3D-CRT: 3-dimensional conformal radiation therapy
ADT: Androgen deprivation therapy
bGS: Biopsy-based gleason score
bPFS: Biochemical progression free survival
cPFS: Clinical progression free survival
DRE: Digital rectal examination
EBRT: External beam radiation therapy
ED2Gy: Equivalent dose at 2 Gy per fraction
GTV: Gross tumor volume
HRs: Hazard ratios
IMRT: Intensity modulated radiation therapy
PCa: Prostate cancer
PCSM: Prostate cancer specific mortality
PCSS: Prostate cancer specific survival
sPFS: Systemic progression free survival
TRUS: Trans-rectal UltraSound

## References

[1] DeSantis CE, Lin CC, Mariotto AB, Siegel RL, Stein KD, Kramer JL (2014). Cancer treatment and survivorship statistics, 2014. CA Cancer J Clin.

[2] D’Amico AV (2011). Risk-based management of prostate cancer. N Engl J Med.

[3] Roach M, Waldman F, Pollack A (2009). Predictive models in external beam radiotherapy for clinically localized prostate cancer. Cancer.

[4] D’Amico AV, Whittington R, Malkowicz SB, Schultz D, Fondurulia J, Chen MH (2000). Clinical utility of the percentage of positive prostate biopsies in defining biochemical outcome after radical prostatectomy for patients with clinically localized prostate cancer. J Clin Oncol Off J Am Soc Clin Oncol.

[5] D’Amico AV, Schultz D, Silver B, Henry L, Hurwitz M, Kaplan I (2001). The clinical utility of the percent of positive prostate biopsies in predicting biochemical outcome following external-beam radiation therapy for patients with clinically localized prostate cancer. Int J Radiat Oncol Biol Phys.

[6] Siddiqui SA, Sengupta S, Slezak JM, Bergstralh EJ, Leibovich BC, Myers RP (2006). Impact of patient age at treatment on outcome following radical retropubic prostatectomy for prostate cancer. J Urol.

[7] Ribeiro M, Ruff P, Falkson G (1997). Low serum testosterone and a younger age predict for a poor outcome in metastatic prostate cancer. Am J Clin Oncol.

[8] Epstein JI, Allsbrook WC, Amin MB, Egevad LL (2005). ISUP Grading Committee. The 2005 International Society of Urological Pathology (ISUP) Consensus Conference on Gleason Grading of Prostatic Carcinoma. Am J Surg Pathol.

[9] Edge S, Byrd D, Compton C, Fritz A, Greene F, Trotti A (2010). AJCC cancer staging manual (7th ed).

[10] Wang JZ, Guerrero M, Li XA (2003). How low is the alpha/beta ratio for prostate cancer?. Int J Radiat Oncol Biol Phys.

[11] Dasu A, Toma-Dasu I (2012). Prostate alpha/beta revisited -- an analysis of clinical results from 14 168 patients. Acta Oncol Stockh Swed.

[12] Valdagni R, Italia C, Montanaro P, Lanceni A, Lattuada P, Magnani T (2005). Is the alpha-beta ratio of prostate cancer really low? A prospective, non-randomized trial comparing standard and hyperfractionated conformal radiation therapy. Radiother Oncol J Eur Soc Ther Radiol Oncol.

[13] Roach M, Hanks G, Thames H, Schellhammer P, Shipley WU, Sokol GH (2006). Defining biochemical failure following radiotherapy with or without hormonal therapy in men with clinically localized prostate cancer: recommendations of the RTOG-ASTRO Phoenix Consensus Conference. Int J Radiat Oncol Biol Phys.

[14] Epstein JI (2011). Prognostic significance of tumor volume in radical prostatectomy and needle biopsy specimens. J Urol.

[15] Algarra R, Zudaire B, Tienza A, Velis JM, Rincón A, Pascual I (2014). Optimizing D’Amico risk groups in radical prostatectomy through the addition of magnetic resonance imaging data. Actas Urol Esp.

[16] Briganti A, Karakiewicz PI, Chun FK-H, Gallina A, Salonia A, Zanni G (2007). Percentage of positive biopsy cores can improve the ability to predict lymph node invasion in patients undergoing radical prostatectomy and extended pelvic lymph node dissection. Eur Urol.

[17] D’Amico AV, Renshaw AA, Cote K, Hurwitz M, Beard C, Loffredo M (2004). Impact of the percentage of positive prostate cores on prostate cancer-specific mortality for patients with low or favorable intermediate-risk disease. J Clin Oncol Off J Am Soc Clin Oncol.

[18] Ricardo Kupka da S, Dall’Oglio MF, Sant’Ana AC, Pontes J, Srougi M (2013). Can single positive core prostate cancer at biopsy be considered a low-risk disease after radical prostatectomy?. Int Braz J Urol Off J Braz Soc Urol.

[19] Maggio A, Panaia R, Garibaldi E, Bresciani S, Malinverni G, Stasi M (2012). Impact of age at diagnosis on overall and disease-free survival in men with prostate cancer following conformal 3D radiation therapy. Tumori.

[20] Rete Oncologica Piemonte | Valle d’Aosta [Internet]. [cited 30 Apr 2015]. Available from: http://www.reteoncologica.it/

[21] Fedeli U, Alba N, Ciccone G, Galassi C, Spolaore P (2007). Re: Trends in radical prostatectomy rates. J Natl Cancer Inst.

