# Supplementary material for: Beyond D’Amico risk classes for predicting recurrence after external beam radiotherapy for prostate cancer: the Candiolo classifier
Source: Radiat Oncol. 2016 Feb 24;11:23. doi: 10.1186/s13014-016-0599-5 (PMC4765202; doi:10.1186/s13014-016-0599-5)

Table 1-S – EUREKA-2 study, Radiotherapy participating centers.

Radiotherapy Center	Town	Total Number of patients	Number of patients included in this paper
FPO-IRCCS Cancer Center of Candiolo	Candiolo	1,195	1,009
Maggiore Hospital, Eastern Piedmont University	Novara	214	200
San Luigi Gonzaga Hospital, University of Torino	Orbassano	76	66
Civile Hospital	Ivrea	962	106
Cardinal Massaia Hospital	Asti	256	220
Degli Infermi Hospital	Biella	235	175
European Institute of Oncology, University of Milano	Milano	379	292
Santa Chiara Hospital, University of Pisa	Pisa	195	174
S. Anna Hospital	Como	143	136
Castelli Hospital	Verbania	121	115
**Total**		**3,776**	**2,493**

Table 2-S – Candiolo classifier table with Hazard Ratio combinations.

initial PSA		Positive Cores %		Age		GS ≤ 6			GS 3 + 4			GS 4 + 3			GS 8			GS 9 - 10		
						0,59	0,59	0,59	0,83	0,83	0,83	0,95	0,95	0,95	1,26	1,26	1,26	1,70	1,70	1,70
						cT1	cT2	cT3-4	cT1	cT2	cT3-4	cT1	cT2	cT3-4	cT1	cT2	cT3-4	cT1	cT2	cT3-4
						0,77	0,93	1,4	0,77	0,93	1,4	0,77	0,93	1,4	0,77	0,93	1,4	0,77	0,93	1,4
PSA < 7	0,63	1-20%	0,67	≥70	0,89	0,17	0,21	0,31	0,24	0,29	0,44	0,27	0,33	0,50	0,36	0,44	0,66	0,49	0,59	0,89
	0,63		0,67	<70	1,12	0,21	0,26	0,39	0,30	0,36	0,55	0,35	0,42	0,63	0,46	0,55	0,83	0,62	0,75	1,13
	0,63	21-50%	0,89	≥70	0,89	0,23	0,27	0,41	0,32	0,39	0,58	0,37	0,44	0,66	0,48	0,58	0,88	0,65	0,79	1,19
	0,63		0,89	<70	1,12	0,29	0,34	0,52	0,40	0,48	0,73	0,46	0,55	0,84	0,61	0,74	1,11	0,82	0,99	1,49
	0,63	51-80%	1,11	≥70	0,89	0,28	0,34	0,51	0,40	0,48	0,72	0,46	0,55	0,83	0,60	0,73	1,10	0,81	0,98	1,48
	0,63		1,11	<70	1,12	0,36	0,43	0,65	0,50	0,60	0,91	0,57	0,69	1,04	0,76	0,92	1,38	1,03	1,24	1,86
	0,63	81-100%	1,5	≥70	0,89	0,38	0,46	0,69	0,54	0,65	0,98	0,62	0,74	1,12	0,82	0,99	1,48	1,10	1,33	2,00
	0,63		1,5	<70	1,12	0,48	0,58	0,87	0,68	0,82	1,23	0,77	0,94	1,41	1,03	1,24	1,87	1,39	1,67	2,52
PSA 7-15	0,96	1-20%	0,67	≥70	0,89	0,26	0,31	0,47	0,37	0,44	0,67	0,42	0,51	0,76	0,56	0,67	1,01	0,75	0,91	1,36
	0,96		0,67	<70	1,12	0,33	0,40	0,60	0,46	0,56	0,84	0,53	0,64	0,96	0,70	0,84	1,27	0,94	1,14	1,71
	0,96	21-50%	0,89	≥70	0,89	0,35	0,42	0,63	0,49	0,59	0,88	0,56	0,67	1,01	0,74	0,89	1,34	1,00	1,20	1,81
	0,96		0,89	<70	1,12	0,43	0,53	0,79	0,61	0,74	1,11	0,70	0,85	1,27	0,93	1,12	1,69	1,25	1,51	2,28
	0,96	51-80%	1,11	≥70	0,89	0,43	0,52	0,78	0,61	0,73	1,10	0,69	0,84	1,26	0,92	1,11	1,67	1,24	1,50	2,26
	0,96		1,11	<70	1,12	0,54	0,65	0,99	0,76	0,92	1,39	0,87	1,05	1,59	1,16	1,40	2,11	1,56	1,89	2,84
	0,96	81-100%	1,5	≥70	0,89	0,58	0,70	1,06	0,82	0,99	1,49	0,94	1,13	1,70	1,24	1,50	2,26	1,68	2,03	3,05
	0,96		1,5	<70	1,12	0,73	0,88	1,33	1,03	1,24	1,87	1,18	1,42	2,15	1,56	1,89	2,84	2,11	2,55	3,84
PSA > 15	1,65	1-20%	0,67	≥70	0,89	0,45	0,54	0,81	0,63	0,76	1,14	0,72	0,87	1,31	0,95	1,15	1,74	1,29	1,56	2,34
	1,65		0,67	<70	1,12	0,56	0,68	1,02	0,79	0,96	1,44	0,91	1,09	1,65	1,20	1,45	2,18	1,62	1,96	2,95
	1,65	21-50%	0,89	≥70	0,89	0,59	0,72	1,08	0,84	1,01	1,52	0,96	1,15	1,74	1,27	1,53	2,31	1,71	2,07	3,11
	1,65		0,89	<70	1,12	0,75	0,90	1,36	1,05	1,27	1,91	1,20	1,45	2,19	1,60	1,93	2,90	2,15	2,60	3,91
	1,65	51-80%	1,11	≥70	0,89	0,74	0,89	1,35	1,04	1,26	1,89	1,19	1,44	2,17	1,58	1,91	2,88	2,13	2,58	3,88
	1,65		1,11	<70	1,12	0,93	1,13	1,69	1,31	1,58	2,38	1,50	1,81	2,73	1,99	2,40	3,62	2,69	3,24	4,88
	1,65	81-100%	1,5	≥70	0,89	1,00	1,21	1,82	1,41	1,70	2,56	1,61	1,95	2,93	2,14	2,58	3,89	2,88	3,48	5,24
	1,65		1,5	<70	1,12	1,26	1,52	2,29	1,77	2,14	3,22	2,03	2,45	3,69	2,69	3,25	4,89	3,63	4,38	6,60

Table 3-S – Candiolo classifier table with complete patients' distribution.

initial PSA	Positive Cores %	Age	GS ≤ 6			GS 3 + 4			GS 4 + 3			GS 8			GS 9 - 10		
			cT1	cT2	cT3-4	cT1	cT2	cT3-4	cT1	cT2	cT3-4	cT1	cT2	cT3-4	cT1	cT2	cT3-4
PSA < 7	1-20%	≥70	86	49	4	19	15	2	5	9	0	5	3	0	0	0	0
		<70	50	39	5	5	5	0	0	2	0	0	0	0	0	0	0
	21-50%	≥70	64	91	6	22	48	5	11	20	1	5	22	2	1	5	6
		<70	24	39	4	7	16	1	2	7	0	2	4	2	0	2	1
	51-80%	≥70	8	13	2	3	13	2	5	4	1	3	7	1	1	3	1
		<70	5	12	0	2	3	2	1	2	1	2	1	1	0	3	3
	81-100%	≥70	4	6	1	2	9	2	0	3	0	0	3	2	0	1	4
		<70	1	11	0	1	1	2	1	0	0	0	4	0	0	2	0
PSA 7-15	1-20%	≥70	74	53	1	18	14	0	6	9	1	9	6	1	1	3	0
		<70	32	18	6	1	4	0	2	2	1	0	1	0	0	0	0
	21-50%	≥70	55	108	10	20	64	6	10	28	3	10	22	5	5	8	1
		<70	24	40	4	4	22	2	4	13	3	1	10	3	0	2	3
	51-80%	≥70	12	23	6	5	24	2	0	18	2	2	8	3	1	6	2
		<70	4	10	1	1	11	3	0	3	2	1	1	2	0	3	2
	81-100%	≥70	5	16	0	3	13	6	4	7	1	2	13	3	1	9	6
		<70	3	8	1	2	3	2	0	3	0	0	1	3	0	4	3
PSA > 15	1-20%	≥70	22	14	0	5	7	1	2	7	0	4	4	1	0	0	0
		<70	6	8	1	1	2	1	2	0	0	1	1	1	0	0	0
	21-50%	≥70	14	25	5	5	13	5	3	14	2	4	16	4	1	8	3
		<70	4	8	2	2	13	3	1	3	1	1	8	6	0	1	0
	51-80%	≥70	1	15	2	3	13	5	1	3	4	2	13	6	1	11	0
		<70	0	9	2	0	12	2	0	7	3	1	2	0	0	2	2
	81-100%	≥70	1	15	2	0	15	6	1	12	3	0	13	8	0	10	14
		<70	0	6	2	0	13	7	0	10	9	0	13	6	1	5	12

Figure 1-S – Graphical assessment of the Proportional Hazard assumption of the Cox model (observed data compared to predicted ones).

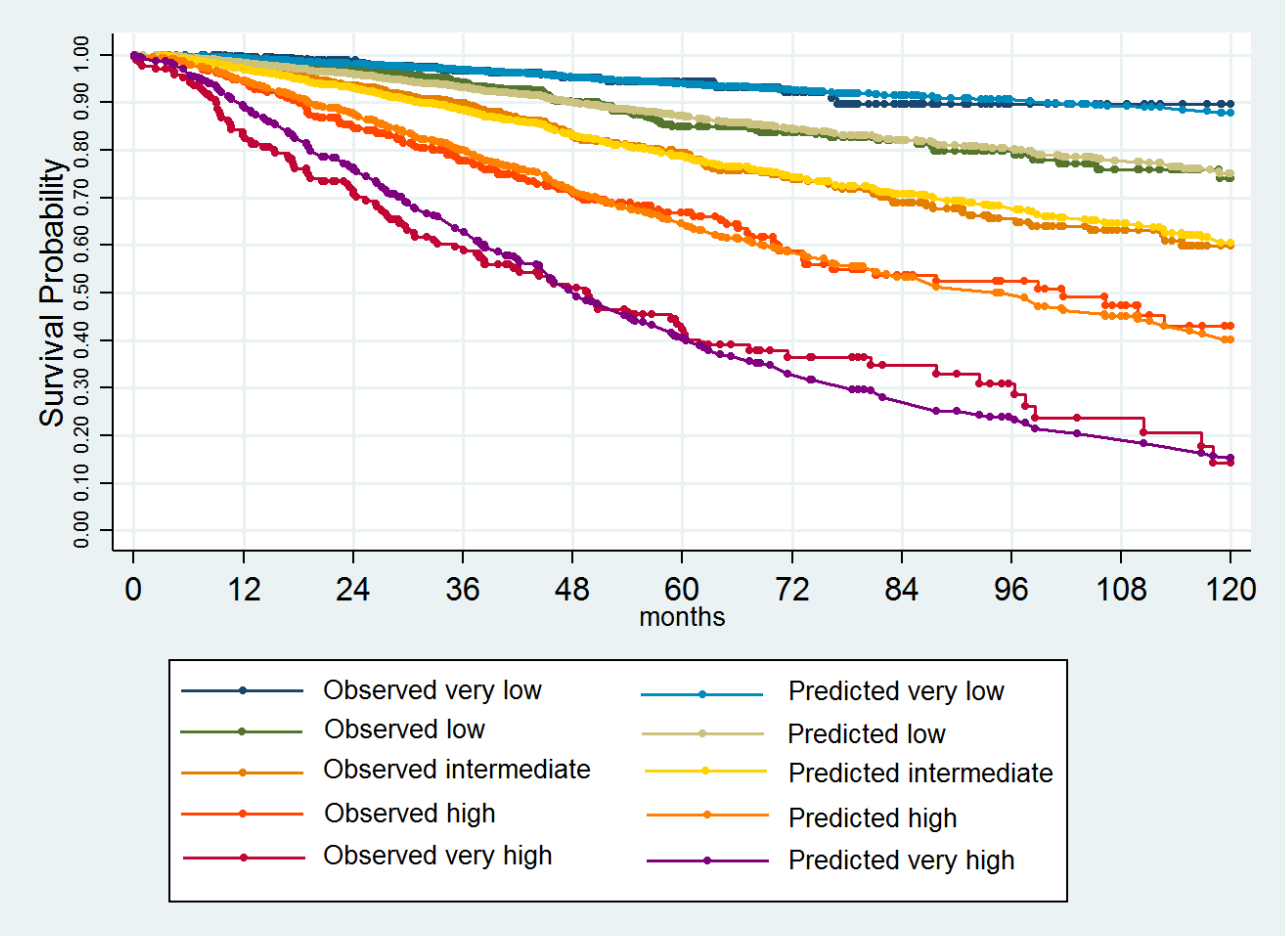

Supplement: Additional file 1: — Table S1. EUREKA-2 study, Radiotherapy participating centers. Table S2. Candiolo classifier table with Hazard Ratio combinations. Table S3. Candiolo classifier table with complete patients’ distribution. Figure S1. Graphical assessment of the Proportional Hazard assumption of the Cox model (observed data compared to predicted ones). (PDF 381 kb) [file 13014_2016_599_MOESM1_ESM.pdf]
